# TRPA1 triggers hyperalgesia and inflammation after tooth bleaching

**DOI:** 10.1038/s41598-021-97040-w

**Published:** 2021-08-31

**Authors:** Chang Chen, Xiansheng Huang, Wenqiang Zhu, Chen Ding, Piaopiao Huang, Rong Li

**Affiliations:** 1 Department of Stomatology, The Second Xiangya Hospital, Central South University, Changsha, Hunan China; 2 Department of Cardiovascular Medicine, The Second Xiangya Hospital, Central South University, Changsha, Hunan China

**Keywords:** Cell biology, Stem cells, Diseases, Pathogenesis

## Abstract

Hyperalgesia has become a major problem restricting the clinical application of tooth bleaching. We hypothesized that transient receptor potential ankyrin 1 (TRPA1), a pain conduction tunnel, plays a role in tooth hyperalgesia and inflammation after bleaching. Dental pulp stem cells were seeded on the dentin side of the disc, which was cut from the premolar buccal tissue, with 15% (90 min) or 40% (3 × 15 min) bleaching gel applied on the enamel side, and treated with or without a TRPA1 inhibitor. The bleaching gel stimulated intracellular reactive oxygen species, Ca^2+^, ATP, and extracellular ATP in a dose-dependent manner, and increased the mRNA and protein levels of hyperalgesia (TRPA1 and PANX1) and inflammation (TNFα and IL6) factors. This increment was adversely affected by TRPA1 inhibitor. In animal study, the protein levels of TRPA1 (*P* = *0.0006*), PANX1 (*P* < *0.0001*), and proliferation factors [PCNA (*P* < *0.0001*) and Caspase 3 (*P* = *0.0066*)] increased significantly after treated rat incisors with 15% and 40% bleaching gels as detected by immunohistochemistry. These results show that TRPA1 plays a critical role in sensitivity and inflammation after tooth bleaching, providing a solid foundation for further research on reducing the complications of tooth bleaching.

## Introduction

Tooth bleaching is one of the most frequently performed clinical procedures in esthetic dentistry^1^, which involves lightening the stained tooth by the application of chemical agents, hydrogen peroxide (H_2_O_2_), or carbamide peroxide, to oxidize the organic pigmentation in the tooth. Despite the high success rate of bleaching of discolored teeth, bleaching sensitivity (BS)^2^ remains the most common clinical side effect related to the bleaching procedure. Studies have reported that the incidence rate of BS reached up to 67–87% in trials^3–5^, and usually persists for up to 4–7 days after bleaching treatment^1^.

The mechanisms underlying BS have not yet been elucidated completely. BS has been suggested to result from a reversible inflammatory process caused by H_2_O_2_ in the dental pulp^6,7^. Several clinical trials have evaluated the use of drugs for the reduction of BS in a perioperative protocol, including anti-inflammatory and analgesic drugs, and desensitizing gels^8^. However, topical application of desensitizers before or after dental bleaching did not reduce the incidence or intensity of BS^8–10^. Thus, it is necessary to further understand the mechanisms of BS, which can facilitate the development of novel strategies with a good efficacy and safety profile to relieve BS.

H_2_O_2_ and its by-products can diffuse through the enamel and dentin, ultimately reaching the pulp to trigger the release of inflammatory mediators^11^, thereby decreasing cellularity and cellular metabolism, altering vascular permeability, and even causing tissue necrosis^7,11^. Increasing evidence suggests that H_2_O_2_-induced pain is associated with transient receptor potential ankyrin 1 (TRPA1)^12,13^. TRPA1 is a non-selective cation channel, which has been recognized as a polymodal nociceptor activated by various irritants and oxidative stimuli, including H_2_O_2_, and contributes to nociceptive and inflammatory pain generation^14,15^. An increased H_2_O_2_ content may contribute to visceral hyperalgesia by activating TRPA1^16^. H_2_O_2_ production and subsequent TRPA1 activation reportedly contribute to the painful and inflammatory responses during acute gout attacks^13^. Another study showed that intramuscular injection of H_2_O_2_ produces nociceptive and aversive behaviors via the TRPA1 receptor, and these H_2_O_2_-induced behaviors were blocked by TRPA1 antagonists^17^. These findings indicate the crucial role of TRPA1 in regulating peroxide-induced pain hyperalgesia, although the mechanism is not yet fully understood.

TRPA1 expression has also been reported in dental pulp cells and odontoblasts^18,19^, and is upregulated in inflamed or injured pulp^18^. The functionality of TRPA1 channels has been shown to be modulated by caries-induced inflammation, indicating a potential mechanism for inflammatory hyperalgesia^20^. Recent studies have demonstrated that mechanically stimulated dentin hypersensitivity after dentin exposure is related to the opening of TRP channels and subsequent activation of the intradental nerve^21^. ATP is released from mechanically stimulated odontoblasts and pulp via pannexin-1 (PANX1) in response to TRP channel activation, which then upregulates the expression of P2X3 receptors on the trigeminal neurons, increasing the intercellular calcium ions of the nerve^21^. Among the large family of TRP channels, TRPA1 and TRPV1 are the best known for their principal role in key hyperalgesia mechanisms^22^. As a small molecule drug that inhibits TRPA1, HC030031 can be used as a tool to study the role of TRPA1 channels in pain^23^.

However, there has been no study on the relationship between the expression and functional significance of TRPA1 in dental pulp during bleaching treatment. In this study, we aimed to elucidate the functional properties of pulpal afferents in the sensory transduction of dentinal pain. The null hypothesis was that bleaching gel does not excite or sensitize the pulpal nociceptors via regulating TRPA1.

## Results

### Dental pulp stem cell (DPSC) characterization

**Figure 1:**
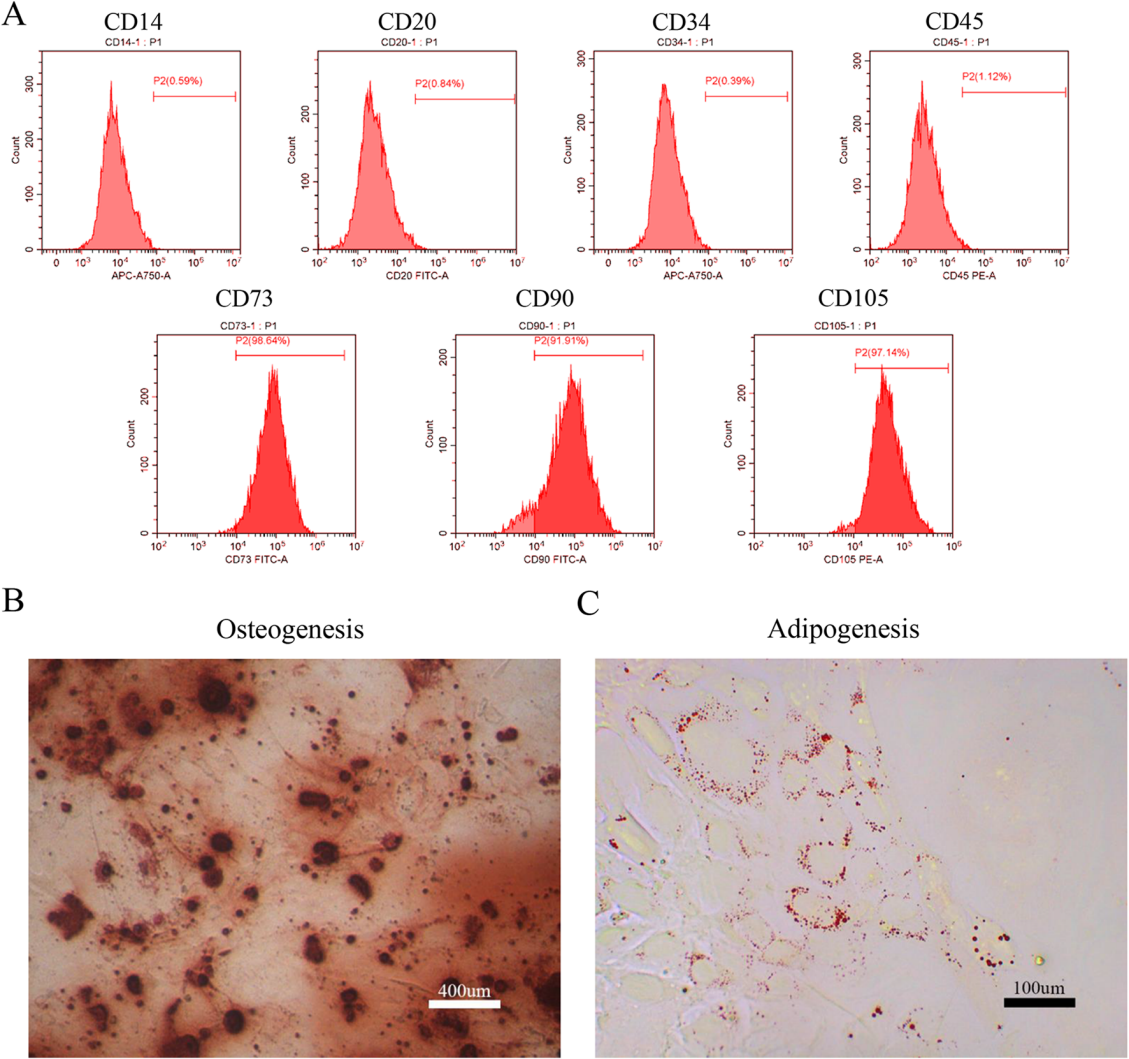
Identification of dental pulp stem cells (DPSCs). Human DPSCs were positive for the cell surface antigens CD73, CD90, and CD105, as well as negative for CD14, CD20, CD34, and CD45 demonstrated by flow cytometry (**A**). DPSCs were cultured under osteogenic (**B**, 14 days) or adipogenic (**C**, 21 days) conditions, and showed mineralized nodules and lipid clusters as revealed by alizarin red and oil red staining, respectively. Scale bar = 400 (**B**) or 100 (**C**) μm.

Flow cytometry showed that human DPSCs were positive for CD73, CD90, and CD105 and negative for CD14, CD20, CD34, and CD45 (Fig. 1A). Moreover, DPSCs showed apparent alizarin red-stained mineralized nodules (Fig. 1B) and oil red O-stained lipid clusters (Fig. 1C).

### TRPA1 triggers inflammation, oxidative stress, and Ca^2+^ influx in DPSCs after bleaching

**Figure 2:**
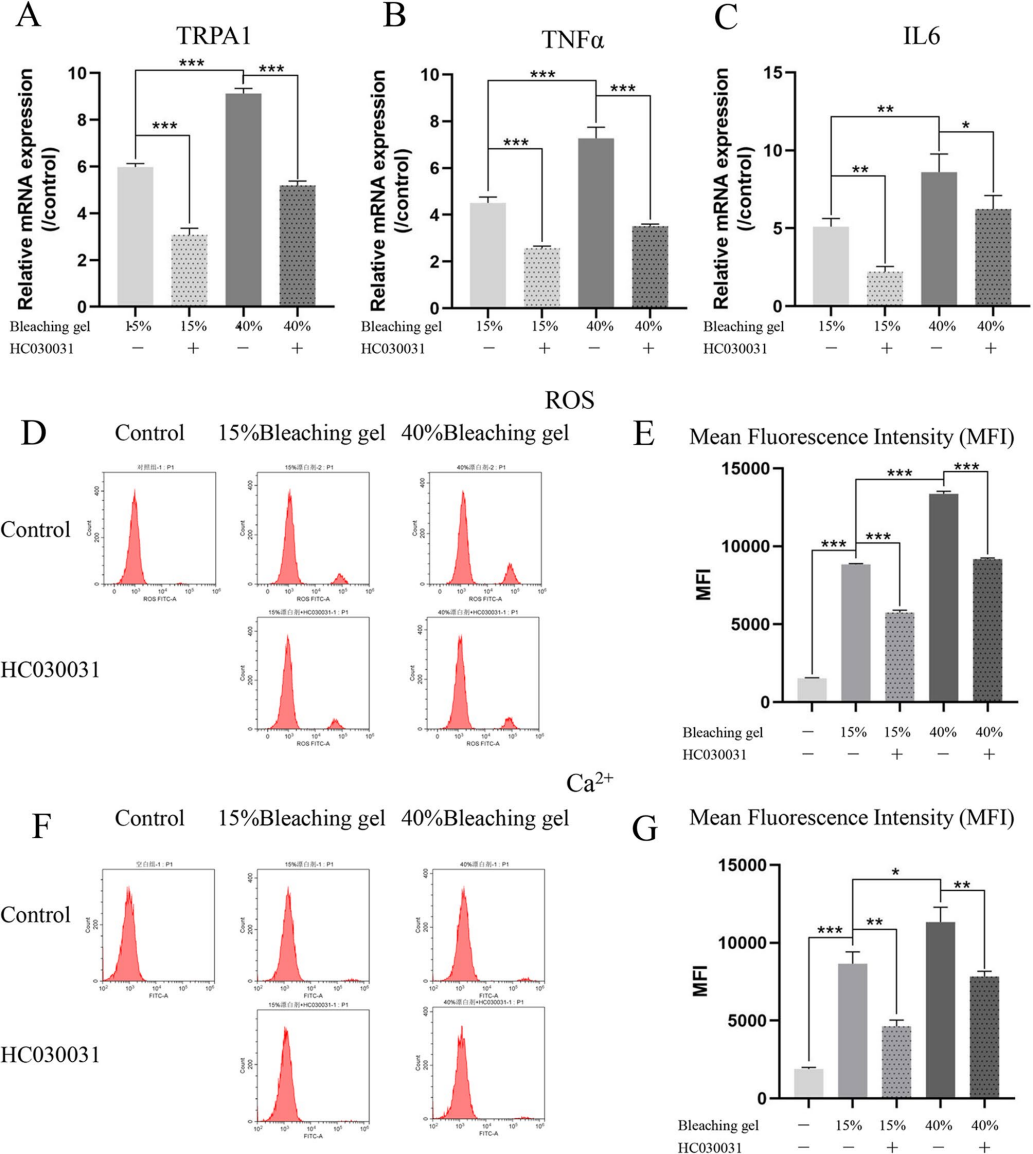
TRPA1triggers inflammation, oxidative stress, and Ca^2+^ influx in DPSCsafter bleaching. RT-PCR for TRPA1 (**A**, Ca^2+^ influx channel), TNFα (**B**), and IL6 (**C**) expression. Bleaching gel induced upregulation of these genes in a dose-dependent manner, whereas HC030031 (TRPA1 inhibitor) reduced this effect. Fluorescence staining and flow cytometry showed that the changes in intracellular ROS (**D** and **E**) and Ca^2+^ (**F** and **G**) were the same as those in TRPA1 mRNA. Data are presented as the mean ± SD, *P < *0.05*, **P < *0.01*, ***P < *0.001*, Student’s *t-*tests. TRPA1, transient receptor potential ankyrin 1; DPSCs, dental pulp stem cells; RT-PCR, reverse transcription-polymerase chain reaction; ROS, reactive oxygen species; MFI, mean fluorescence intensity.

To investigate the significance of the Ca^2+^ influx channel TRPA1 in BS, DPSCs were treated with the TRPA1 inhibitor HC030031 after 15% and 40% bleaching treatment. Reverse transcription-polymerase chain reaction (RT-PCR) showed that TRPA1 mRNA expression increased significantly in a dose-dependent manner after the bleaching gel was applied on the disc, which slightly recovered upon the addition of HC030031 (Fig. 2A, *P* < *0.0001*). A similar significant increase in the mRNA levels of the inflammatory factors TNFα (15% *vs.* 40% *P* = *0.0009*; 15% *vs.* 15% + HC030031 *P* = *0.0002*; 40% *vs.* 40% + HC030031 *P* = *0.0002*) and IL6 (15% *vs.* 40% *P* = *0.0089*; 15% *vs.* 15% + HC030031 *P* = *0.0013*; 40% *vs.* 40% + HC030031 *P* = *0.0475*) was observed after application of the bleaching gel (Fig. 2B,C). A bleach concentration-dependent significant increase and reversal by HC030031 were also observed in the levels of oxidative stress (Fig. 2D,E, *P* < *0.0001*) and Ca^2+^ influx (Fig. 2F,G; Control *vs.* 15% *P* = *0.0001*; 15% *vs.* 40% *P* < *0.019*; 15% *vs.* 15% + HC030031 *P* = *0.0013*; 40% *vs.* 40% + HC030031 *P* = *0.0038*) using the reactive oxygen species (ROS) fluorescent dye H_2_DCFDA and the Ca^2+^ fluorescent dye fluo-3AM, respectively, which were quantified by flow cytometry.

### TRPA1 induces the hyperalgesia pathway in DPSCs after bleaching

**Figure 3:**
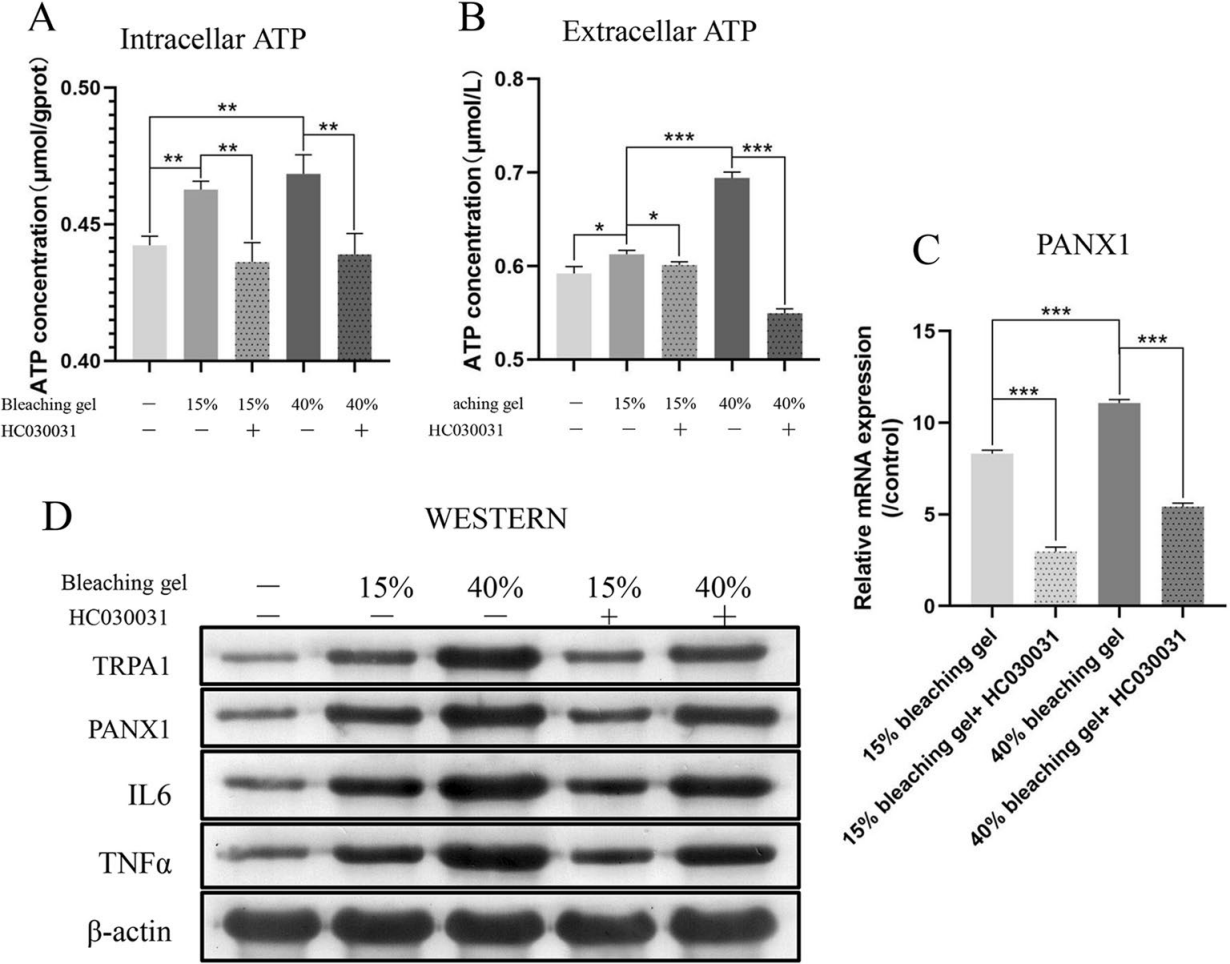
TRPA1 induces the hyperalgesia pathway in DPSCs after bleaching. Intracellular (**A**) and extracellular (**B**) ATP levels increased after bleaching, and the TRPA1 inhibitor HC030031 reversed this trend. The mRNA expression of pannexin-1 (PANX1) (**C**), ATP exocytosis channel, and protein expression of TRPA1, PANX1, IL6 and TNFα, detected by western blotting (**D**), matching the changes in ATP. Full-length blots/gels are presented in Supplementary Fig. S4. Data are presented as the mean ± SD, *P < *0.05*, **P < *0.01*, ***P < *0.001*, Student’s *t-*tests.

In the hyperalgesia pathway, ATP is released in large quantities in cells, and the amount of ATP flowing out of the cells by the PANX1 channel is also increased to act on nerve cells, causing pain after an increase in intracellular Ca^2+^. Thus, we measured intracellular (Fig. 3A; Control *vs.* 15% *P* = *0.0015*; 15% *vs.* 40% *P* = *0.2665*; 15% *vs.* 15% + HC030031 *P* = *0.0041*; 40% *vs.* 40% + HC030031 *P* = *0.0080*) and extracellular (Fig. 3B; Control *vs.* 15% *P* = *0.0156*; 15% *vs.* 40% *P* < *0.0001*; 15% *vs.* 15% + HC030031 *P* = *0.0225*; 40% *vs.* 40% + HC030031 *P* < *0.0001*) ATP levels after bleach gel application. ATP levels increased in a bleach gel dose-dependent manner, which was restored by the addition of HC030031, in line with the changes in the expression of PANX1 mRNA (Fig. 3C; 15% *vs.* 40% *P* < *0.0001*; 15% *vs.* 15% + HC030031 *P* = *0.0013*; 40% *vs.* 40% + HC030031 *P* = *0.0475*). Among them, the increase between the 15% and 40% gels was not significantly different. We speculate this is caused by the dynamic balance of synthesis and decomposition of ATP in the cell. Western blotting showed similar changes in the protein levels of TRPA1 (15% *vs.* 40% *P* = *0.0001*; 15% *vs.* 15% + HC030031 *P* = *0.0188*; 40% *vs.* 40% + HC030031 *P* = *0.0004*), PANX1 (15% *vs.* 40% *P* < *0.0001*; 15% *vs.* 15% + HC030031 *P* < *0.0001*; 40% *vs.* 40% + HC030031 *P* = *0.0249*), TNFα (15% *vs.* 40% *P* = *0.0004*; 15% *vs.* 15% + HC030031 *P* = *0.0483*; 40% *vs.* 40% + HC030031 *P* = *0.0018*), and IL6 (15% *vs.* 40% *P* = *0.0352*; 15% *vs.* 15% + HC030031 *P* = *0.0262*; 40% *vs.* 40% + HC030031 *P* = *0.0481*) to those at the mRNA level (Fig. 3D). The statistical results obtained using Quantity One software are displayed in Fig. S2.

### Immunohistochemical analysis

**Figure 4:**
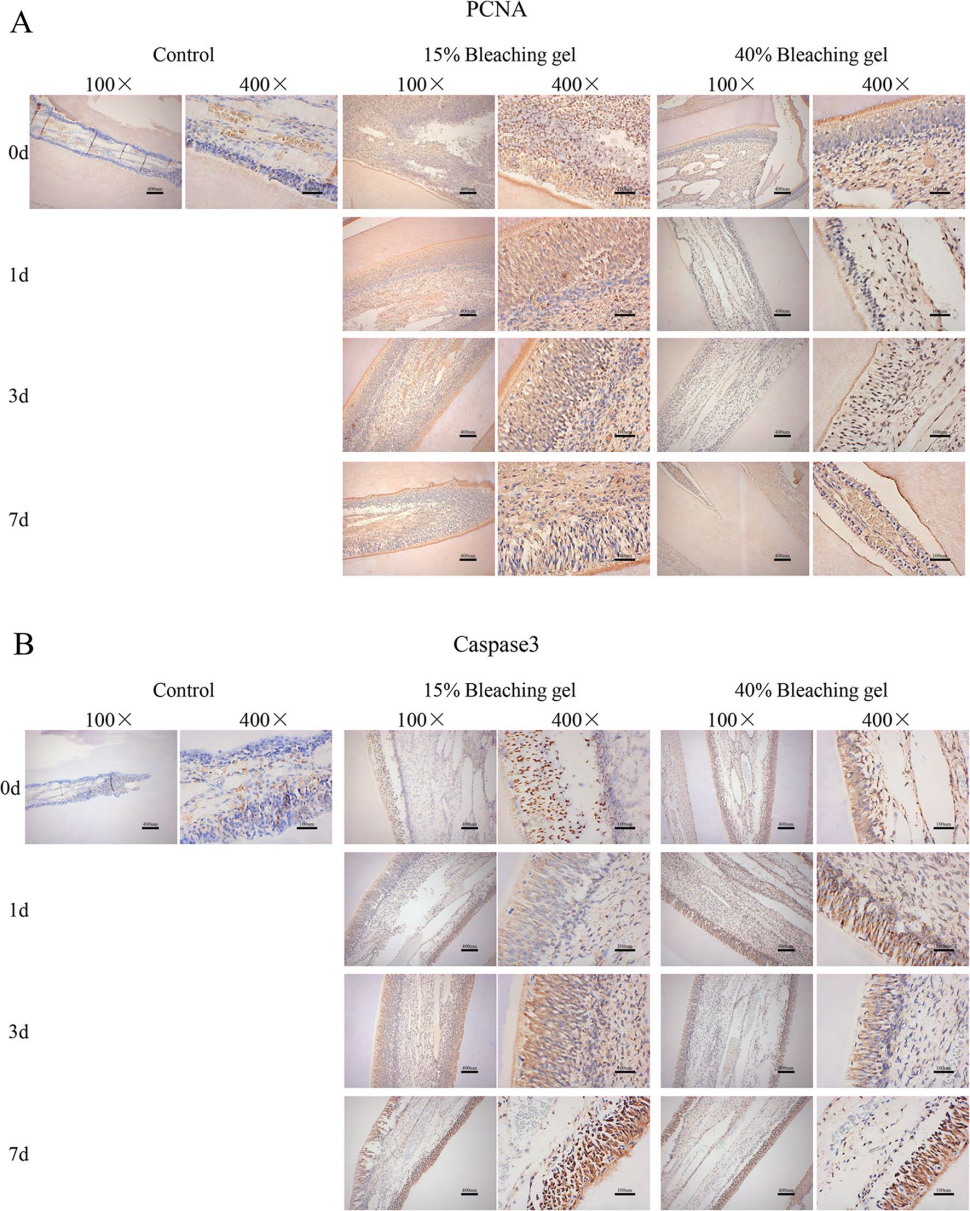
Changes in proliferation-related proteins after bleaching in a rat incisor model. Immunohistochemistry revealed the expression of proliferative factors, PCNA (**A**) and Caspase3 (**B**), in the incisor pulp of the rat. Bleaching significantly increased the expression of these factors in vivo, and the 15% gel demonstrated stronger enhancement. Bars = 400 μm in 100× magnification and 100 μm in 400× magnification.

**Figure 5:**
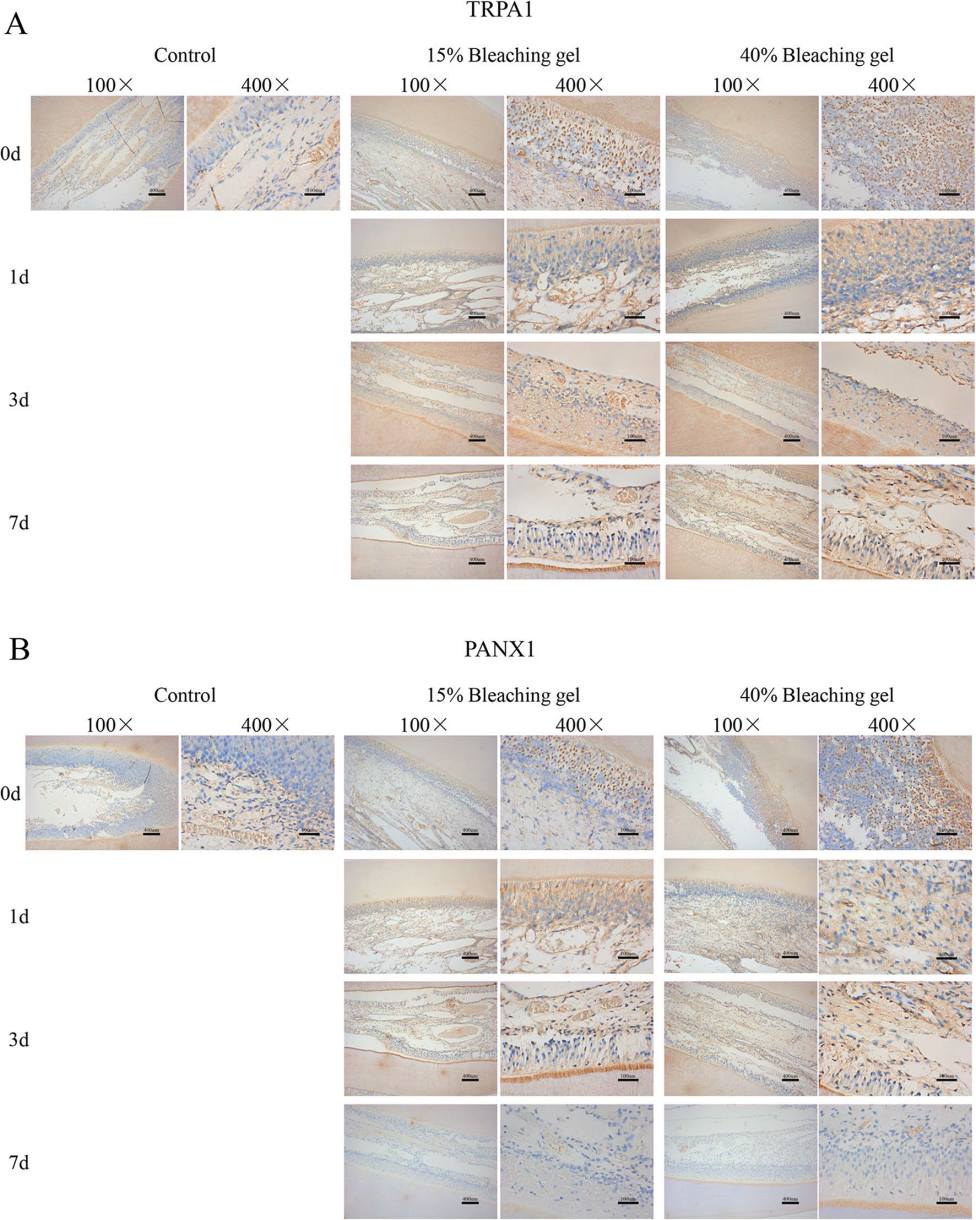
Changes in the factors causing hyperalgesia after bleaching in a rat incisor model. TRPA1 (**A**) and PAXN1 (**B**) protein expression in the incisor pulp measured by immunohistochemistry. Bleaching gel increased their expression and the 40% gel exhibited stronger staining. Decrease in cellularity, cellular disorganization especially in odontoblast layer are shown in bleached incisor pulp of 0 d. Bar = 400 μm in 100× magnification and 100 μm in 400× magnification.

We then established a rat incisor model to examine the changes in the in vivo pulp within seven days after applying low (15%) and high (40%) concentrations of the bleaching gel. The proliferation-related proteins PCNA (Fig. 4A) and caspase 3 (Fig. 4B) were assessed using immunohistochemistry. The low-concentration group displayed stronger staining than that in the control and high-concentration groups, indicating that the bleaching gel promoted proliferation, but a high concentration weakened this ability. In addition, the hyperalgesia factors TRPA1 (Fig. 5A) and PANX1 (Fig. 5B) were sufficiently expressed in the control group, which was attributed to the stimulation from tooth extraction. However, the bleaching gel further increased the TRPA1 and PANX1 protein expression in the pulp. The quantitative result of the mean integrated optical density (IOD) is shown in Supplementary Fig. S3 (TRPA1 *P* = *0.0006*, PANX1 *P* < *0.0001*; PCNA *P* < *0.0001* and caspase 3 *P* = *0.0066*). In terms of histological response, compared with the normal pulp in the control group, the bleached pulp tissue showed a decrease in cellularity and cellular disorganization, especially in the odontoblast layer.

## Discussion

With the increasing use of tooth bleaching technology, its complications have also attracted considerable attention, particularly BS. Understanding the underlying mechanism is essential to reduce the incidence of BS and promote clinical application of this procedure. To this end, we conducted a series of experiments to explore the effect of inhibiting TRPA1, a key factor in hyperalgesia, on sensitivity to bleaching gel. Our results suggest that the bleaching gel evidently upregulated TRPA1 expression in DPSCs, thereby activating the hyperalgesia gene PANX1 and the inflammatory genes TNFα and IL6 in a concentration-dependent manner. Profound changes in the protein levels of TRPA1, PANX1, TNFα, IL6, and the proliferation-related factors PCNA and caspase 3 were found in bleaching gel-treated teeth. Therefore, we must reject our initial hypothesis.

In the sensory pathway, a receptor perceives the stimulation and then delivers a neurotransmitter, which in turn stimulates the nerve. Therefore, the sensor pathway is composed of receptors, neurotransmitters released by receptors, and nerves. Shibukawa et al.^21^ immunohistochemically explored the receptors, TRPA1 channels that perceive the stimulation and PANX1 channels that secrete ATP as a neurotransmitter in the pulp tissue, making use of specific antibodies targeting these channels and nerves during odontoblast differentiation. TRPA1 is considered to be a receptor for harmful cold temperatures^24^, and its mRNA expression has been reported to increase under the influence of inflammation in the mouse colon^25^. TRPA1 expression is upregulated in the trigeminal ganglion after injection of nerve growth factor^26^ or due to pulpal inflammation^27^. Moreover, TRPA1 expression is elevated in the odontoblasts of carious teeth^20^. Although the mechanism of dentin hyperalgesia remains unclear, the significant activation of protein expression in many TRP channels in the pulp or DPSCs suggests that TRP channels, specifically TRPA1, have a vital sensory function and play an important role in the pathogenesis of dentin hyperalgesia^28^. TRP channels in DPSCs can be switched on by the corresponding stimuli, thus participating in sensing and responding to various stimuli to ultimately induce hyperalgesia. Therefore, TRPA1 could be crucial to the dentin hyperalgesia induced by mechanical, thermal, or chemical stimulation.

Supporting this hypothesis, we found that bleaching gel enhanced the oxidative stress of DPSCs, based on an increase in intracellular ROS levels. The stimulation receptor TRPA1 was activated, promoting the transport of calcium ions into cells, therefore sensitizing PANX1 to secrete ATP into the extracellular zone. As mentioned above, ATP acts as a neurotransmitter to stimulate neurons and produce pain. Tooth bleaching produced inflammation, causing pulpitis in some teeth^29^. Based on the mechanism underlying inflammation, the inflammatory response and complications of pulpitis can be attenuated.

The cytoplasmic extension of odontoblasts and the dentin fluid acts as a physical barrier for the penetration of foreign substances, minimizing the damage to the dental pulp^30,31^. Therefore, an in vivo model is of great significance in studying the response of the dental pulp tissue to invaders. Studies on human teeth^32^, animal teeth^33^, and cell cultures^34^ have shown that the pulp of bleached teeth exhibits dramatic changes on the first day after bleaching, manifesting as severe inflammation or necrosis. Another study^35^ showed that the concentration of the bleaching gel affects the ability of H_2_O_2_ to penetrate the pulp cavity and is therefore related to the extent of injury in the pulp. In the present study, we found apparent expression of TRPA1, PANX1, and proliferation-related proteins in the pulp after bleaching, especially in odontoblasts, which was consistent with the in vitro results. However, anterior teeth, which are the teeth most commonly bleached in clinical contexts, are rarely obtained; consequently, the enamel/dental disk in this study was made from human premolars. This issue needs to be addressed in future research.

**Figure 6:**
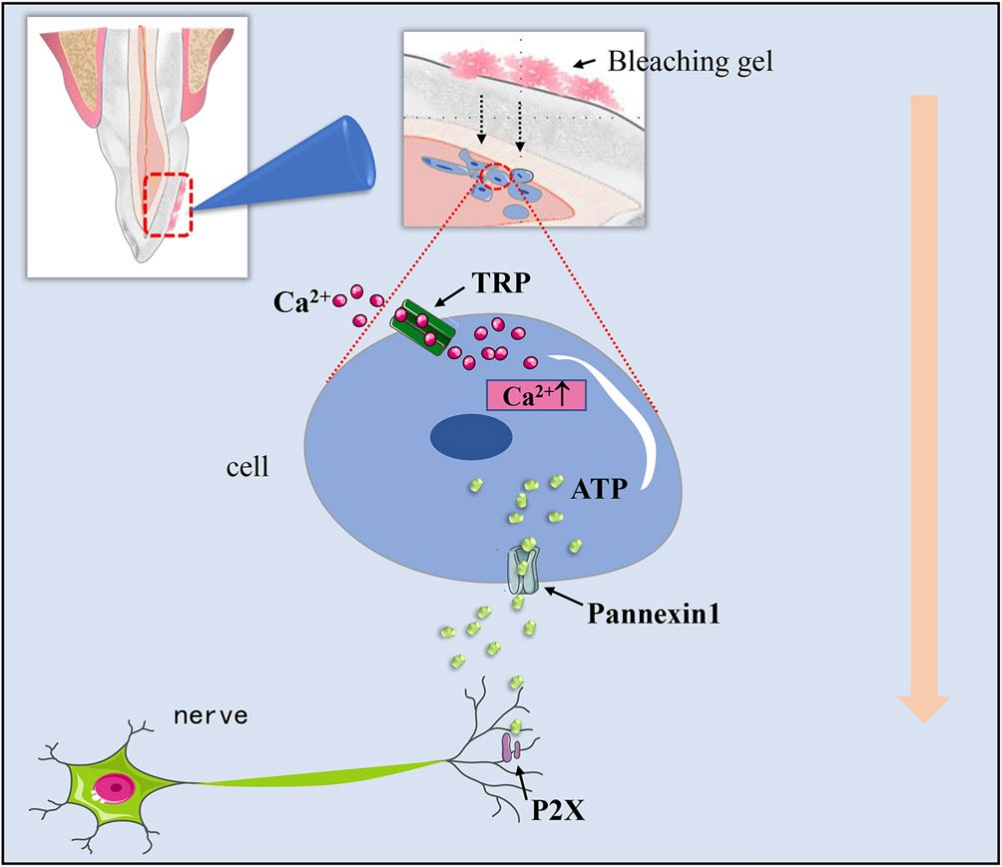
Schematic diagram presenting the hypothesis on the role of TRPA1 in bleaching sensitivity. Tooth bleaching leads to the activation of TRPA1, which promotes the intracellular absorption of calcium. In turn, PANX1 is activated, which induces the secretion of ATP that acts as a neurotransmitter and induces pain.

In conclusion, we demonstrated that tooth bleaching leads to the activation of TRPA1, which promotes the intracellular absorption of calcium. In turn, PANX1 is activated, which induces the secretion of ATP that acts as a neurotransmitter to ultimately induce pain (Fig. 6). In addition, TRPA1 upregulates TNFα and IL6, thus promoting the occurrence of inflammation. This study thus clarifies the role of TRPA1 in the development of hyperalgesia and inflammation after bleaching, laying a solid foundation for further research on reducing tooth bleaching complications and promoting its clinical application.

## Methods

### Cell culture

DPSCs were selected as representative cells for this study because they are the progenitor cells of all dental pulp cells and are easily available. Human DPSCs were obtained from clinically healthy extracted teeth of patients aged 18–24 years, with their consent and the approval of the ethics committee of the Second Xiangya Hospital, Central South University, China [March 10, 2017; Ethical Review No. (Research 146)]. After extraction, the pulp was cut into small pieces (approximately 1 mm^3^) and treated for 24 h in complete Dulbecco’s modified Eagle’s medium (DMEM, Sigma Aldrich; Merckgroup, St Louis, Missouri [MO], US) supplemented with 100 IU/mL penicillin (Beyotime; Beyotime Shanghai, China), 100 mg/mL streptomycin (Beyotime), 2 mmol/L glutamine (Gibco; Thermo Fisher Scientific, Waltham, Massachusetts [MA], US), containing 10% fetal bovine serum (FBS, Gibco; Thermo Fisher Scientific, Waltham, Massachusetts [MA], US) and 200 units/mL of type II collagenase (Worthington Biochemical Corporation; Worthington Biochemical Corporation, New Jersey [NJ], US). The resolved cells were incubated in DMEM supplemented with 10% FBS. The cells at the third passage were used in the subsequent experiments as described previously^36^ and cultured on the dentin surface of the disc. Informed consent was obtained from all participants in this study, and this study was performed in line with the principle of Declaration of Helsinki.

### Cell identification

#### Identification of stem cell surface markers

Trypsin (Beyotime) was used to detach the cells, which were then counted and aliquoted in eight Eppendorf tubes (1.5 mL, 1 × 10^5^ cells/tube). One tube was used as a negative control group and only had phosphate buffered saline (PBS) containing 2% FBS added to it. Then, 100 μL of PBS containing flow cytometry antibody and 2% FBS was added to the other seven tubes, which were incubated at 4 °C in the dark for 30 min. After that, the tube was centrifuged (1000 rpm, 5 min) followed by resuspension in 200 μL PBS. A flow cytometer was used to detect the cells (BD Biosciences; BD Biosciences, San Jose, California [CA], US) The FL1 channel was used for detecting CD20-FITC (11-0209-41, Thermo Fisher Scientific, eBioscience, San Diego, California [CA], US), CD73-FITC (11-0739-41, Thermo Fisher Scientific, eBioscience) and CD90-FITC (11-0909-41, Thermo Fisher Scientific, eBioscience). The FL2 channel was used for detecting CD45-PE (12-0459-41, Thermo Fisher Scientific, eBioscience) and CD105-PE (12-1057-41, Thermo Fisher Scientific, eBioscience). The FL4 channel was used for detecting CD14-APC (17-0149-41, Thermo Fisher Scientific, eBioscience) and CD34-APC (17-0349-41, Thermo Fisher Scientific, eBioscience).

#### Adipogenesis

Briefly, 1 × 10^6^ DPSCs were inoculated in a six-well plate, and 2 mL of complete medium was added to each well. The medium was changed every 3 days until the cell confluence reached 100%. The adipogenic differentiation basal medium (Cyagen Biosciences; Cyagen Biosciences, Guangzhou, Guangdong, China) was used. Specifically, the complete DMEM was discarded, and 2 mL of stem cell adipogenic differentiation medium A (liquid A) was added to the six-well plate. After 3 days of induction, liquid A was drawn from the six-well plate, and 2 mL of human umbilical cord mesenchymal stem cell adipogenic differentiation medium B liquid (liquid B) was added to the six-well plate. After 24 h, liquid B was removed, and liquid A was added for induction. This cycle was repeated five times, and then, liquid B was left in the six-well plate for 5 days to maintain the culture. During this maintenance period, liquid B was changed with fresh liquid B every 2 days. After the cells became saturated, the six-well plate was washed twice with PBS, and the cells were fixed with 4% paraformaldehyde (Sigma Aldrich; Merckgroup, St Louis, Missouri [MO], US) for 30 min. The six-well plate was washed with PBS twice, and 7 mM of Oil Red O (Sigma Aldrich; Merckgroup) dye solution was added at room temperature for 30 min to stain the cells, which were then washed with PBS three times. Pictures were then taken under a microscope (Cnmicro; Cnmicro, Beijing, China).

#### Osteogenesis

DPSCs were inoculated in a six-well plate, and 2 mL of complete medium was added to each well. When the cell confluence reached approximately 70%, the complete medium was drawn from the well, and 2 mL of the osteogenic medium (Cyagen Biosciences; Cyagen Biosciences, Guangzhou, Guangdong, China) was added to the six-well plate. The induction medium was refreshed every 3 days. After 25 days of induction, the six-well plate was washed twice with PBS, and the cells were fixed with 4% paraformaldehyde (Sigma Aldrich, Merckgroup, St Louis, Missouri [MO], US) for 30 min. The six-well plate was washed with PBS twice, and 7 mM of Oil Red O (Sigma Aldrich, Merckgroup) dye solution was added at room temperature for 30 min to stain the cells, which were then washed with PBS three times. Pictures were then taken under a microscope (Cnmicro).

### Enamel/dentin disc preparation

Complete premolars were obtained from orthodontic patients aged 18–24 years with their consent. The inclusion criteria were as follows: (1) age, 18–24 years; (2) premolars had to be removed owing to orthodontics; and (3) the premolars were healthy, complete, and untreated. The exclusion criteria were as follows: (1) patients with systemic diseases, such as infectious, autoimmune, and genetic diseases and (2) those with abnormal tooth structure, such as dental fluorosis. Dental crowns were obtained by horizontally sectioning the tooth at the cementoenamel junction using a diamond saw (MCTBIO; MCTBIO, Yongin, Gyeonggi, Korea). The lingual sections of the crowns were discarded to fabricate a buccal enamel-dentin disc (Fig. S1). The apical wall was then sealed using light-cured resin (Filtek P60; 3M ESPE, St Paul, Minnesota [MN], US) to produce a bottomless cylinder. The specimens were disinfected with 75% ethanol (Aladdin; Aladdin, Shanghai, China) and ultraviolet light irradiation for 1 h before the in vitro experiments^11^. After disinfection, grouping was performed by simple randomization; that is, samples were numbered in sequence and then divided into groups.

### Experimental design

Cells were implanted on the dentin side of the disc. After attachment, the enamel-dentin disc was overturned in the medium to expose the enamel surface above the liquid level with a 1 mm gap; the enamel surface was surrounded by a light cured resin barriers (Opalescence PF; Ultradent Products, South Jordan, Utah [UT], US) to prevent the bleach from spilling into the medium (Fig. S1). After that, approximately 0.5 mL of thickened bleaching gel was daubed on the enamel surface at 37 °C. We tested two concentrations of bleaching gel, 40% H_2_O_2_ and 15% carbamide peroxide (Opalescence Boost and Opalescence PF, respectively; Ultradent Products, South Jordan, Utah [UT], US)^6^. Five groups were created: G1, untreated DPSCs; G2, 15% bleaching gel for 90 min; G3, 15% bleaching gel and 1 μM HC030031 (TRPA1 inhibitor, ApexBio; ApexBio, Houston, Texas [TX], US) for 90 min; G4, 40% bleaching gel for three 15-min periods in a row; and G5, 40% bleaching gel and 1 μM HC030031 for three 15-min periods in a row. As a small molecule drug that inhibits TRPA1, HC030031 can be used as a tool to study the role of TRPA1 channels in pain^23^. In the inhibition group, HC030031 was dissolved in the culture medium to directly inhibit TRPA1.

### Oxidative stress assay

Oxidative stress was detected using a 5 mM cell-permeant fluorescence probe, 20,70-dichlorodihydrofluorescein diacetate (H_2_DCFDA) (Life Technologies; Thermo Fisher Scientific, Carlsbad, California [CA], US). After the different treatments mentioned above, DPSCs were incubated with H_2_DCFDA for 30 min at 37 °C. Then, the cells were washed three times with serum-free cell culture medium to fully remove H_2_DCFDA that did not enter the cells. Trypsin was used to detach the cells, which were then resuspended in PBS and tested using flow cytometry in the FL1 channel^37^, with a minimum of 6000 events acquired per sample.

### Real-time PCR

The mRNA expression levels of *TNF*α, *TRPA1*, *IL-6*, and *PANX1* were measured using real-time PCR as previously described^11,36^. Total RNA was extracted using TRIzol (Invitrogen; Thermo Fisher Scientific, Waltham, Massachusetts [MA], US), which was then reverse-transcribed to single-stranded cDNA using the HiFiScript cDNA Synthesis Kit (CWBIO; CWBIO, Beijing, China). QPCR was performed by initial incubation at 95 °C for 2 min, and then at 95 °C for 5 s and 60 °C for 30 s for 40 cycles. The housekeeping gene actin and the 2^−ΔΔct^ method were used to normalize the relative expression levels. Table 1 presents the primer sequences (Sangon; Sangon, Shanghai, China).

**Table 1:** Primer sequences.

Gene	Forward (5′ to 3′)	Reverse (5′ to 3′)
Actin	ACCCTGAAGTACCCCATCGAG	AGCACAGCCTGGATAGCAAC
*TNF*α	GAACCCCGAGTGACAAGCCT	TATCTCTCAGCTCCACGCCAT
*IL6*	GCAATAACCACCCCTGACCCAA	GCTACATTTGCCGAAGAGCC
*TRPA1*	GCATGTTTATTCCCTCACTACCCC	ACACAAGGACACATACATAGCCA
*PANX1*	ACTTGGTTTCCCCGCATGGT	GAACAAAGCGCTTCCCTCTGG

### Western blotting

Western blotting was performed as previously described^38^. In brief, proteins were resolved by sodium dodecyl sulfate–polyacrylamide gel electrophoresis using a Bio-Rad system (Bio-Rad; Bio-Rad, Hercules, California [CA], US) and 15% acrylamide gel. The proteins were then transferred onto nitrocellulose membranes (Millipore; Merckgroup, Burlington, Massachusetts [MA], US). The membrane was blocked with 10% non-fat dry milk (Applygen; Applygen, Beijing, China) in 25 mM Tris-buffered saline (pH 7.2, Sigma Aldrich; Merckgroup, St Louis, Missouri [MO], US) and 0.1% Tween 20 (TBST, Sinopharm; Sinopharm, Shanghai, China) for 2 h, followed by incubation with the primary antibodies against TNFα (1:1000, ab215188, Abcam; Abcam, Cambridge, UK), TRPA1 (1:1000, NB110-40763, Novus Biologicals; Novus Biologicals, Colorado [CO], US), PANX1 (1:2000, 12595-1-AP, Proteintech; Proteintech, Rosemont, Illinois [IL], US), IL6 (1:1000, ab233706, Abcam; Abcam, Cambridge, UK), and β-actin (1:5000, 60008-1-Ig, Proteintech;Proteintech, Rosemont, Illinois [IL], US). The membrane was washed with TBST and incubated with horseradish peroxidase-conjugated goat anti-rabbit IgG (1:5000, SA00001-2, Proteintech; Proteintech, Rosemont, Illinois [IL], US) at 37 °C for 1 h. The signal bands were visualized by enhanced chemiluminescence using a WesternBright ECL kit (Advansta; Advansta, San Jose, California [CA], US). Blotting intensities were determined using Quantity One software v4.6.6 (BIO-RAD; BIO-RAD, Hercules, California [CA], US) and normalized to the internal control β-actin.

### Intracellular Ca^2+^ measurements

Cellular Ca^2+^ was stained using the fluorescence dye fluo-3AM (Beyotime; Beyotime, Shanghai, China) and analyzed by flow cytometer. The DPSCs were removed from the cell culture solution, and 5 mM fluo-3AM was added. This was then incubated for 20 min in a 37 °C cell incubator. The cells were washed three times with serum-free cell culture medium to fully remove residual staining solution and then detected using a flow cytometer in the FL1 channel.

### ATP measurements

Intracellular and extracellular ATP contents were detected using an ATP Content Assay Kit (Jiancheng; Jiancheng, Nanjing, Jiangsu, China) following the manufacturer protocol. Creatine kinase catalyzes the reaction of creatine and ATP to produce creatine phosphate. The optical density (OD) value can be detected by the phosphomolybdic acid colorimetric method at 700 nm, which is directly proportional to the ATP content. The OD value was measured with a microplate reader (Heales; Heales, Shenzhen, Guangdong, China) after combining the reagents given in the manufacturer instructions. The cell protein content was detected using a bicinchoninic acid assay kit (HonorGene; HonorGene, Changsha, Hunan, China) simultaneously. Finally, the intracellular ATP concentration was calculated using Eq. (1):

1 $$\left( {{\text{OD}}_{{{\text{test}}}} - {\text{OD}}_{{{\text{control}}}} /{\text{OD}}_{{{\text{standard}}}} - {\text{OD}}_{{{\text{blank}}}} } \right) \times {\text{ ATP Content}}_{{{\text{standard}}}} \times {\text{Dilution times}}/{\text{protein content}}_{{{\text{test}}}} .$$

The extracellular ATP was calculated using Eq. (2):

2 $$\left( {{\text{OD}}_{{{\text{test}}}} - {\text{OD}}_{{{\text{control}}}} /{\text{OD}}_{{{\text{standard}}}} - {\text{OD}}_{{{\text{blank}}}} } \right) \times {\text{ATP Content}}_{{{\text{standard}}}} \times {\text{Dilution times}}.$$

### Animal experiments

Our study followed the ARRIVE guidelines and National Institutes of Health Guide for the Care and Use of Laboratory Animals (NIH Publications No. 8023, revised 1978). The experimental procedure was approved by the Ethics Committee of the Second Xiangya Hospital, Central South University, China (March 10, 2017, no. 2017005).

Thirty-six Sprague–Dawley rats weighing 250–350 g were kept in a standard animal room at 22 ± 1 °C, with 55% ± 10% humidity and a standard light/dark schedule of food and water. The rats were assigned to one of the following three groups: (1) no bleaching gel (n = 12); (2) 15% bleaching gel for 90 min (n = 12); and (3) 40% bleaching gel for three 15-min periods (n = 12). Bleaching gel (0.01 mL) was painted onto the incisor surface according to the relevant manufacturer instructions. Then, the rats were reared according to the aforementioned standards. Following bleaching, after 0 (n = 3), 1 (n = 3), 3 (n = 3), and 7 (n = 3) days of standard feeding, the rats were sacrificed under deep anesthesia with pentobarbital (80 mg/kg, Sigma Aldrich; Merckgroup, St Louis, Missouri [MO], US).). The incisors were fixed in 4% neutral buffered formalin (Sigma Aldrich; Merckgroup, St Louis, Missouri [MO], US) for 24 h and demineralized in 10% ethylenediaminetetraacetic acid solution (Sigma Aldrich; Merckgroup, St Louis, Missouri [MO], US) for 3 months. The samples were then embedded in paraffin, and sections (3 mm thick) were used as specimens.

### Immunohistochemistry

Immunohistochemistry was performed using an indirect immunoperoxidase technique for PCNA (1:100, ab29, Abcam; Abcam, Cambridge, UK), Caspase3), caspase 3 (1:100, 9664 T, Cell Signaling Technology; Cell Signaling Technology, Beverly, Massachusetts [MA], US), TRPA1 (1:50, NB110-40763, Novus Biologicals; Novus Biologicals, Colorado [CO], US), and PANX1 (1:50, 12595-1-AP, Proteintech; Proteintech, Rosemont, Illinois [IL], US).). The reacted secondary antibodies were visualized using a DAB kit (ZSGB-BIO; ZSGB-BIO, Beijing, China). The analyzes were performed by a single qualified operator in a blinded manner under a light microscope (Leica; Leica, Wetzlar, Germany). Image ProPlus 6.0 software (Media Cybernetics, Inc.; Media Cybernetics, Inc., Rockville, Maryland [MD], USA) was used to determine the mean IOD (IOD/area).

### Statistical analysis

The experiments were repeated at least three times, and the data are presented as the mean ± SD. Statistical analyzes were performed using GraphPad Prism 6.0 (GraphPad Software; GraphPad Software, San Diego, California [CA], US) by TWO-way ANOVA test (immunohistochemistry) or unpaired Student’s *t-*tests (other data). Graphs were plotted using GraphPad Prism 6.0. The level of statistical significance was set at *P* < 0.05.

## Supplementary Information

Supplementary Figures.

## Data Availability

The datasets generated during and/or analyzed during the current study are available from the corresponding author on reasonable request.
