# Supplementary material for: TRPA1 triggers hyperalgesia and inflammation after tooth bleaching
Source: Sci Rep. 2021 Aug 31;11:17418. doi: 10.1038/s41598-021-97040-w (PMC8408176; doi:10.1038/s41598-021-97040-w)
Supplement: Supplementary file 1 — Supplementary Figures. [file 41598_2021_97040_MOESM1_ESM.pdf]

TRPA1 triggers hyperalgesia and inflammation after tooth bleaching

Chang Chen[1†], Xiansheng Huang[2†], Wenqiang Zhu[2], Chen Ding[2], Piaopiao Huang[2], Rong Li[1,]*

[1] *Department of Stomatology, The Second Xiangya Hospital, Central South University, Changsha, Hunan, China*

[2] *Department of Cardiovascular Medicine, The Second Xiangya Hospital, Central South University, Changsha, Hunan, China*

[*]*Correspondence:* rongli@csu.edu.cn

[†]*Equal contributors.*

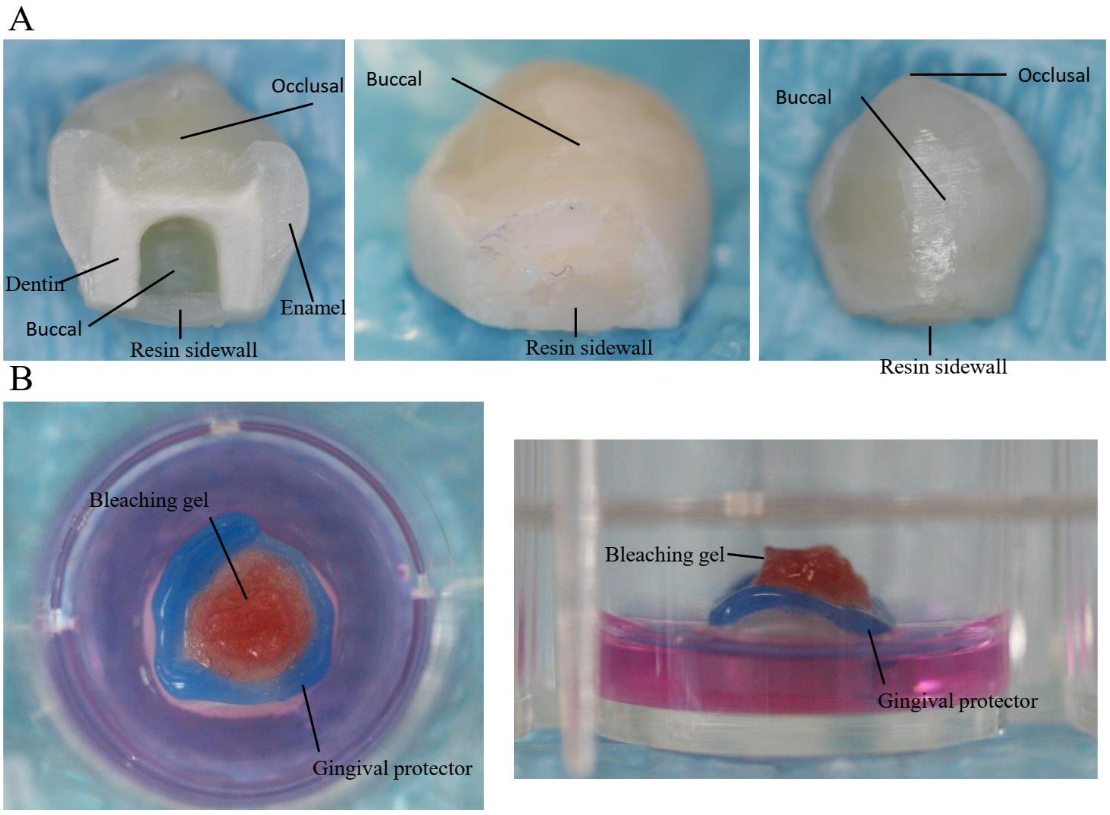

Figure S1. Photos of enamel/dentin disk(A) and bleached sample (B).

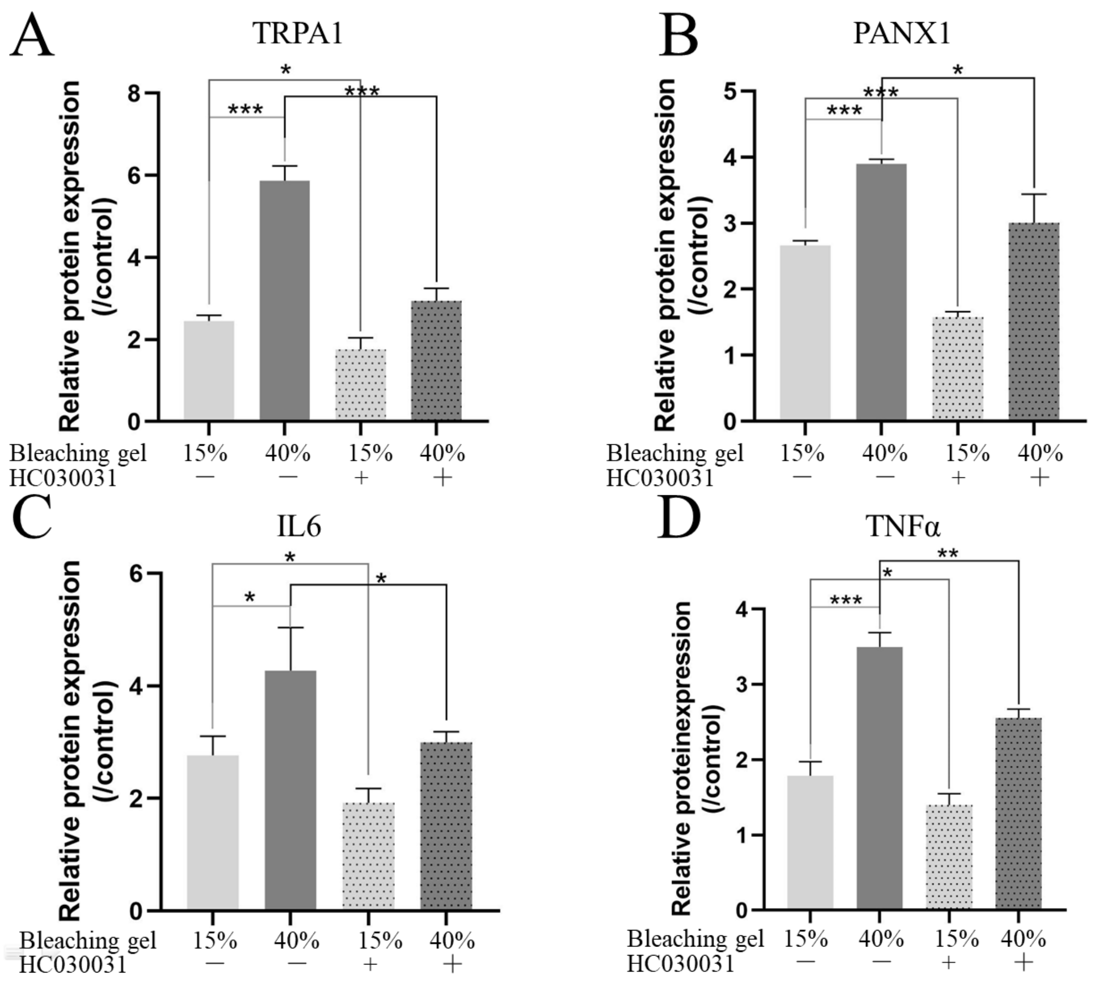

*Figure S2. Quantitative results of western blot. Data are presented as the mean ± SD, *P < .05, **P < .01, ***P < .001,* Student's *t*-tests.

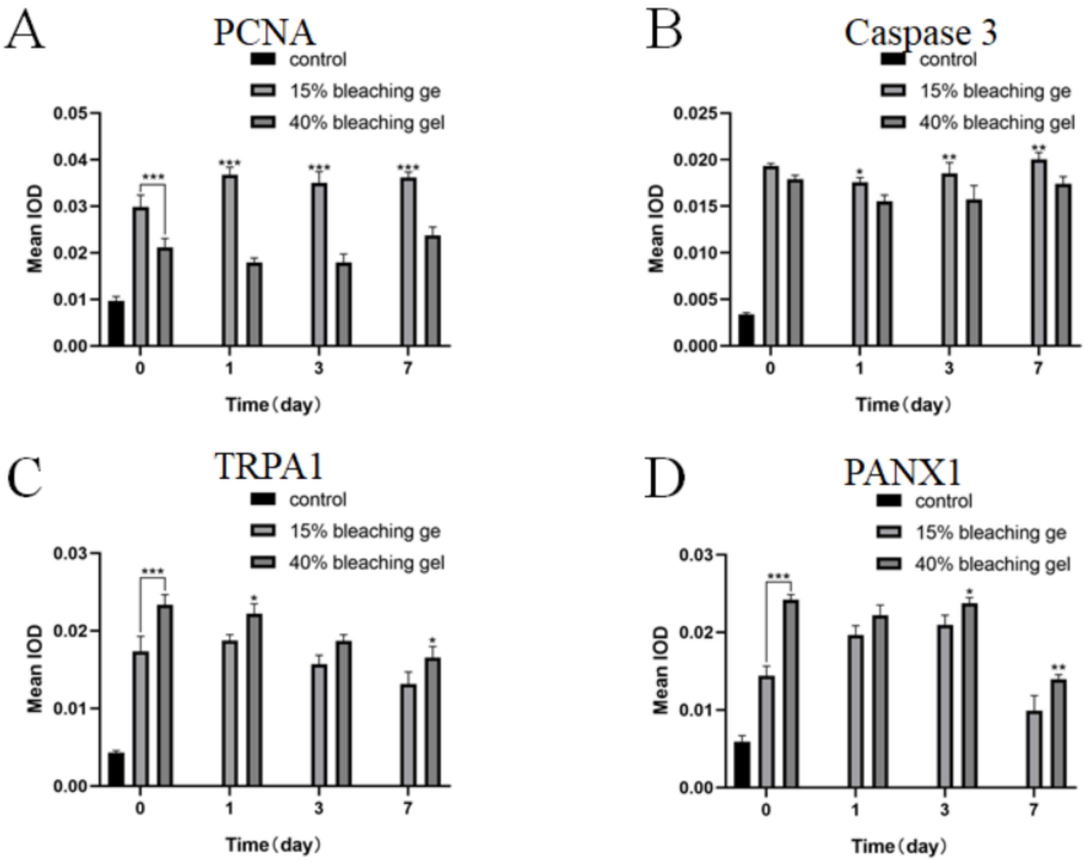

*Figure S3. Immunohistochemistry mean IOD statistical results. Image pro plus was used to detect the mean IOD by means of IOD/area. Data are presented as the mean ± SD, *$P < .05$, **$P < .01$, ***$P < .001$, TWO-way ANOVA test.*

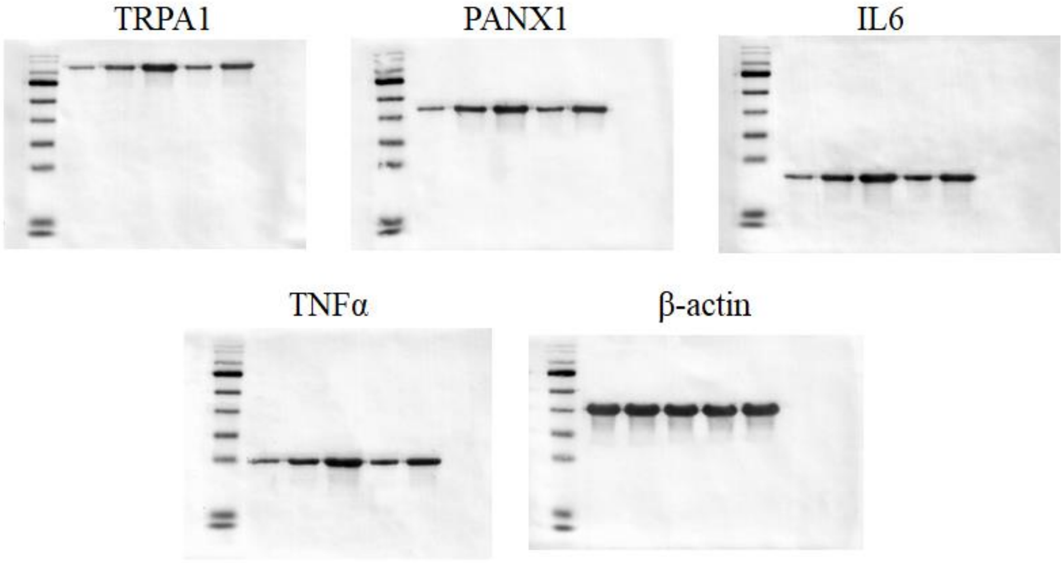

Figure S4. Uncropped western original image.
